# Syncopation creates the sensation of groove in synthesized music examples

**DOI:** 10.3389/fpsyg.2014.01036

**Published:** 2014-09-16

**Authors:** George Sioros, Marius Miron, Matthew Davies, Fabien Gouyon, Guy Madison

**Affiliations:** ^1^Department of Informatics Engineering, Faculdade de Engenharia, Universidade do PortoPorto, Portugal; ^2^Institute for Systems and Computer Engineering - Technology and Science (INESC-TEC)Porto, Portugal; ^3^Department of Psychology, Umeå UniversityUmeå, Sweden

**Keywords:** groove, syncopation, movement, rhythm, listening experiment, music

## Abstract

In order to better understand the musical properties which elicit an increased sensation of wanting to move when listening to music—groove—we investigate the effect of adding syncopation to simple piano melodies, under the hypothesis that syncopation is correlated to groove. Across two experiments we examine listeners' experience of groove to synthesized musical stimuli covering a range of syncopation levels and densities of musical events, according to formal rules implemented by a computer algorithm that shifts musical events from strong to weak metrical positions. Results indicate that moderate levels of syncopation lead to significantly higher groove ratings than melodies without any syncopation or with maximum possible syncopation. A comparison between the various transformations and the way they were rated shows that there is no simple relation between syncopation magnitude and groove.

## Introduction

Certain types of music induce the desire in humans to tap their feet, move their body, and dance. This feeling is commonly known as “groove” and has a strong affective component, as well as a strong correlation with music appreciation (Madison, 2006). According to Janata et al. (2012), we find pleasurable the music that makes us want to dance and such music elicits spontaneous rhythmic movements. Furthermore, Madison et al. (2011) show that the propensity to move differs substantially from one piece of music to another, but can be linked to certain rhythmical properties of the music signal such as beat salience and the density of events.

There is a strong agreement in the previous studies toward the definition of groove. Madison (2006) defined groove as the “sensation of wanting to move some part of the body in relation to some aspect of the sound pattern.” Similarly, Janata et al. (2012) conducted a survey involving a wide variety of music related terms, to find that “groove is that aspect of the music that induces a pleasant sense of wanting to move along with the music.” Furthermore, there are strong clues that link certain properties of the music with the induced groove. Janata et al. (2012) conducted an extensive study on sensorimotor coupling which provides a broad analysis under the different facets of groove. The computational part of the analysis suggests that music genre and faster tempi have a strong effect on the desire to move along to the music. In comparison, Madison et al. (2011) claim that tempo alone cannot explain groove. Consequently, Madison et al. (2011) found that low level descriptors, such as beat salience, event density and fast metrical levels as physical attributes of the sound, highly correlate with groove. In contrast and unlike what musicological literature points toward (Keil, 1995), microtiming does not increase groove. The latter was confirmed by a systematic analysis conducted by Davies et al. (2013) on simple rhythms.

Madison and Sioros (2014) examined how musicians create groove when they are confined to a monophonic melody and an isochronous beat. Professional musicians were asked to perform six simple and six more complex melodies in two different expressive ways: maximizing and minimizing groove. Their performances were then rated by listeners to examine to what extent the manipulations of the musicians created the intended effect. The study revealed that musicians tended to shift the onset and offset positions of the notes in the simple melodies to faster metrical levels in order to increase groove in their performances, and that this increased the groove to the same level as the complex melodies. By virtue of shifting note onsets and offsets to faster metrical levels, the musicians created a greater number of off-beat events, thus, increasing the syncopation. When they tried to minimize groove they were unable to decrease it below the dead-pan level for the simple performances, but the dead-pan level of groove in the complex melodies could be reduced to that of the simple melodies. This seemed to be achieved by two different means. One was to simplify the rhythmic structure toward higher metrical levels, i.e., from 16th and 8th-notes to 8th, 4th, and half-notes, and the other was to break the rhythm such that the sense of a regular beat disappeared. Both of those devices result in decreased syncopation but each in a different way. The first simply eliminates the syncopating notes by placing them on the beat. In the second, syncopation disappears together with the metrical feel.

While the results from Madison and Sioros (2014) appear to indicate that the main physical correlate of groove was syncopation, the design of the experiments was not fully controlled, since the musicians could, and did, change several aspects of their performances simultaneously. It is therefore difficult to determine if the changes in some performance parameters were redundant, independent, or interdependent, and also the unique effect of each one of them. Therefore, there is a need to experimentally test the hypothesis that syncopation *per se* induces the sensation of groove independently of other expressive or structural features of the performances.

Syncopation as a cognitive mechanism has been described as a form of violation of metrical expectations (Huron, 2006, p. 297), that is, temporal expectations that are established in the listener's mind as a function of the temporal regularities in the music signal. Several operational definitions of syncopation are based on the more general expectation principle, and provide algorithms for measuring the amount of syncopation present in a rhythmic pattern (Longuet-Higgins and Lee, 1984; Gómez et al., 2005; Huron and Ommen, 2006; Sioros and Guedes, 2011). A common characteristic among many syncopation measures is the use of a metrical template that describes the strong and weak beats found in the musical meter. Normally, a note articulated in a weak metrical position is followed by a note in the following strong position resulting in a weak-strong rhythmic figure. Huron (2006, p. 295) suggests that when the expected note on the strong position does not occur, the tension increases and the feeling of syncopation is evoked. The syncopation is then attributed to the “improper binding” of a musical event found on a weak metrical position to an event in the following strong position, which is delayed or not present at all (see also London, 2012, p. 107). Different kinds of weightings related to the strength of the metrical positions have been used in order to quantify syncopation (see Gómez et al., 2007 for various syncopation metrics).

In the context of syncopation and its relation to groove, a very recently published study (Witek et al., 2014) demonstrated a listener preference for moderate syncopation in drum patterns. The study was made using funk drum-breaks with varying degrees of syncopation that were taken in their majority from real music tracks (36 out of 50). The degree of syncopation was then measured using a variant of the algorithm proposed by Longuet-Higgins and Lee (1984). As syncopation has been often associated with rhythmic tension and rhythm complexity (Huron, 2006, p. 295), the study concluded that gradually increasing the syncopation also increases the desire to move, but only up to a certain point beyond which desire decreases.

Additionally, the pleasure experienced when listening to music is often correlated with the violation of expectations (Huron, 2006, p. 297). As a component of musical rhythm, syncopation is a way to create metrical tension (Temperley, 1999), owing to the fact that it occurs only in contradiction with an established metrical pattern (Longuet-Higgins and Lee, 1984), thus breaking an existing expectation. Correspondingly, moving from very simple to more intricate rhythm patterns can elicit different kind of responses from people in terms of enjoyment and desire to move.

In this paper, we focus on syncopation as the physical property of the music signal that can drive the perception of groove. As we want to eliminate all other expressive factors of a musical performance, we developed a computer algorithm that generates syncopation in monophonic sound sequences relying on changing metrical positions only, without altering other expressive or structural characteristics. The algorithm is an adaptation of the general syncopation transformations developed by Sioros et al. (2013). While in the recent Witek study (2014) syncopation was measured in pre-existing drum loops, in our study we can generate and vary it in piano melodies using an automatic algorithm over which we have complete control.

We designed two listener experiments using simple piano melodies that were transformed in an automatic and controlled way in order to generate syncopation without affecting other rhythmic or structural characteristics. In the first experiment we explore the more general hypothesis that increasing the syncopation to a moderate amount and introducing faster metrical levels results in an increased groove sensation. Then, in the second experiment, we aim to gain more insight on how the strength of each individual syncopation felt in a melody influences groove as well as distinguishing between different ways of introducing faster metrical levels. Parallel to the syncopation transformations, density transformations that increase the number of events in the melodies are used as controls in order to verify the effect of syncopation.

Based on recent results on this topic (Madison and Sioros, 2014; Witek et al., 2014), our predictions for the outcome of the listening experiments are: (1) the deadpan versions of the melodies that contain no syncopation would be rated lowest in groove, (2) introducing a small number of strongly syncopating notes would be rated highest in groove, (3) an increased number of syncopated notes would not have a proportional increase in groove ratings or would be rated lower.

## Experiment 1

### Methods

#### Participants

Twenty-eight participants (14 female, 14 male, mean = 30.0 years, *SD* = 2.9 years) of different nationalities were recruited through advertisement on Umeå university campus in Sweden and INESC-TEC in Portugal. They received a remuneration for their participation of 75 Kronor at Umeå university or 10€ at INESC-TEC.

#### Stimuli

**Figure 1:**
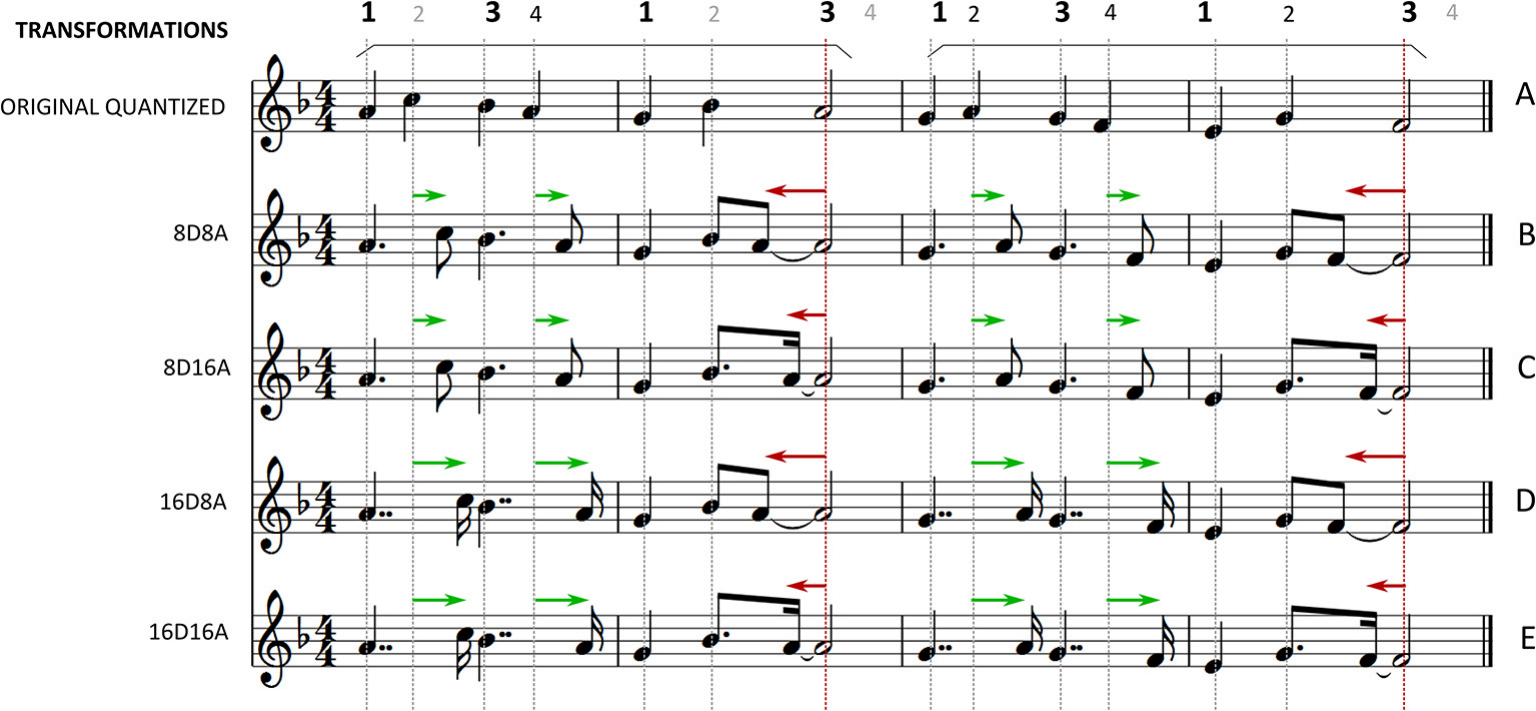
**Example of a piano melody and all the applied transformations of experiment 1**. The beats are annotated above the staffs. **(A)** The original quantized melody. An arch marks the duration of each phrase of the melody (in this example: the first two bars and the last two bars). **(B)** 8D8A. The last “strong” note of each phrase is anticipated by an 8 note. The “weak” quarter notes are shifted to the 8 note position preceding the following strong beat. **(C)** 8D16A. The last “strong” note of each phrase is anticipated by a 16 note. The “weak” quarter notes are shifted to the 8 note position preceding the following strong beat. **(D)** 16D8A. The last “strong” note of each phrase is anticipated by an 8 note. The “weak” quarter notes are shifted to the 16 note position preceding the following strong beat. **(E)** 16D16A. The last “strong” note of each phrase is anticipated by a 16 note. The “weak” quarter notes are shifted to the 16 note position preceding the following strong beat.

Our aim was to measure and understand the effect of syncopation on the sensation of groove, independently of other structural or expressive factors such as the meter or timing deviations from the metronomical positions. At the same time we wanted to eliminate any expressive features of human performances, such as timing deviations from the metrical grid or dynamic accents. To this end, we used simple piano melodies with a clear metrical structure. All melodies were in 4/4 meter and consisted of short melodic phrases of 2–4 bars as in the example of Figure 1. The melodies contain simple rhythmic figures such as the ones found in melodies for children and were of moderate tempo (120 BPM).

Seven melodies were traditional songs for children from different cultures. Another five were composed for the purposes of the experiment. The traditional melodies had a more complex structure compared to the composed ones, with higher variety of phrase lengths, phrase boundaries positions and harmonic and melodic content. A set of simple transformations was then applied to them resulting in 5 total versions for each.

The original melodies included only metrical levels equal to or slower than the quarter note, i.e., quarter notes (q.n. = 500 ms), half notes (1000 ms) and whole notes (2000 ms). They contained no syncopation (Figure 1, staff **A**) and were all quantized so that their timing did not deviate from the metronomic positions. To each melody, we applied a set of transformations in order to introduce syncopation (Figure 1, staffs **B–E**). While the original version contained only slower metrical levels, applying any of the transformations introduced the 8 note metrical level (250 ms) and/or the 16 note metrical level (125 ms).

Before applying any transformation, the melodies were manually segmented into short phrases of 2–4 bars. To this end, the following procedures were applied. The boundaries of the melodic phrases were chosen according to note durations. When, after a long note, i.e. a half or whole note, a series of quarter notes begins, the first quarter note marks the beginning of a phrase. While this rule would be insufficient to define phrase boundaries in more complex melodies, it is valid and efficient for the simply structured phrases of the music examples (MEs) used in this experiment. The transformations were then applied on each phrase separately.

The MIDI files of all melodies and their respective variations are available as Supplementary material.

***Syncopation transformations***. The transformations employ the principles of the “improper binding” of notes articulated in weak metrical positions to missing notes in the following strong positions in order to generate syncopation (London, 2012, p. 107). First, the meter is divided in strong and weak beats. Then, the last note event of each phrase is syncopated by anticipating it to an earlier weaker metrical position. The last note of each phrase is a half or whole note articulated on a strong beat, as a direct consequence of the way the melodic phrases were segmented. The anticipated offbeat notes are tied to the following silent strong beats where they originally belonged giving rise to the feeling of syncopation. Therefore, the expectation of a note onset occurring at the strong beat is violated and the established meter is momentarily contradicted (Temperley, 1999; Huron, 2006, p. 295; Huron and Ommen, 2006).

The syncopation transformations aim to introduce syncopation in a way that the perceived meter and other rhythmical and structural characteristics of the melodies are preserved. In order not to affect the perceived meter, the remainder of the notes on strong beats, i.e., all notes on the first and third quarter note of each bar besides the last one of each phrase, were kept in their original positions. However, the notes found in the weak positions, i.e., on the second and fourth quarter note, were delayed so that the previous note, articulated on a strong beat, is now lengthened and therefore felt as accented (Povel and Okkerman, 1981). In this way, the meter is first established by the notes on the strong beats at the beginning of each phrase. Then, the anticipated note is placed at an offbeat weak position and is tied to the following strong beat where it originally belonged (Figure 1, staffs **B–E**) generating syncopation. The note articulated in the weak position before the last note of each phrase is not delayed in order to leave space for the syncopating note to be anticipated. The delay of the weakly articulated notes introduces faster metrical levels in addition to the metrical levels introduced by the syncopation.

Four different syncopation transformations were applied on the original melodies that differ on the metrical subdivisions that the displaced notes were found after the transformation. They are coded by two numbers, *x* and *y*, in the following form *xDyA*, where *x* denotes the metrical level that the weak quarter notes were shifted to and *y* is the metrical level where the last note of each phrase is shifted to. For example, in the *16D8A* transformation the notes originally articulated on weak quarter notes were shifted to the 16 note position directly preceding the next strong beat, while the last note of each phrase in the melodies was shifted to the preceding 8 note metrical position (Figure 1, staff **D**).

#### Implementation of musical examples

To generate all the versions for each melody, the original melodies were imported as MIDI files in Ableton Live where they were quantized and edited in order not to contain durations shorter than quarter notes. Simple Max for Live devices were developed in order to automatically apply the corresponding transformations. MIDI files were generated for all 6 versions of each melody and were then rendered into 16 bit wave files using high quality piano samples^1^.

#### Rating scales

The experiment was intended to explore the “movement inducing” effect of syncopation. To this end, participants were asked to rate groove while listening to musical stimuli. Groove was defined as “the sensation of wanting to move some part of your body in relation to some aspect of the music.” However, the participants were told that the purpose of the experiment was the general study of rhythm perception, so that the response bias was minimized. For this reason two additional rating scales were included in the experiment, namely familiarity and preference.

All three scales were presented to participants under the global question “How well do the following words describe your experience of the music?” The ratings ranged from 0 for “not at all” to 10 for “entirely” in unit increments. A separate horizontal slider initially positioned at 5 was available for entering the ratings for each scale. The scales were presented in the same order throughout the experiment.

#### Design

The dependent variable was the rating of groove, and the independent variables were (1) the type of transformation of the melodies (5 levels including 1 quantized and 4 different types of syncopation), (2) melody type (simple or complex), and (3) the melodies themselves. A within-participants design was employed in which each participant rated all 60 conditions (5 transforms × 12 melodies), which were presented in a different random order to each participant. Randomization and other functionality was built into a custom-made presentation and response recording software.

#### Procedure

The participants listened to the music examples alone through headphones in a quiet room and rated them on a computer. First, a paper with instructions that included the definitions of the rating scales was given to them. Prior to the beginning of the listening test, they were asked if the task and terminology was clear. After signing the informed consent form, a training session preceded the experiment proper, in which the participants rated two music examples which allowed them to set the volume to a comfortable level and to familiarize themselves with the program and the procedure. The sliders were locked while the music was playing to force the participants to listen to the entire example before rating. After rating each example the participant could continue to the next by clinking on a button. The entire session lasted between 30 and 40 min.

### Results

**Figure 2:**
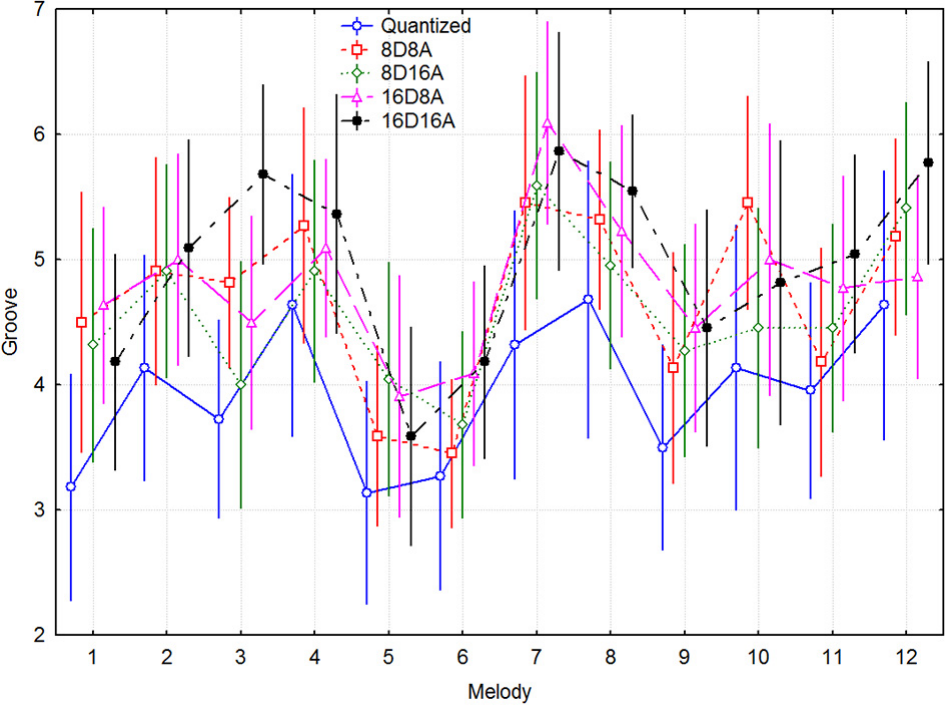
**Mean groove ratings for transform and melody, across participants**. Melodies 1–5 are the simple ones and melodies 6–12 are the complex traditional songs.

Since there were a different number of melodies in each group, this precluded a full-factorial model. A within-participants 5 transformations × 5 melody 2-Way ANOVA demonstrated main effects for the simple melodies of transform [*F*_(4, 84)_ = 6.678, *p* < 0.0005] and melody [*F*_(4, 84)_ = 11.56, *p* < 0.000001], but not of their interaction [*F*_(16, 336)_ = 1.604, *p* = 0.066]. The complex melodies exhibited a similar pattern of effects, according to a 5 transformations × 7 melody 2-Way ANOVA. There was a main effect of transform [*F*_(4, 84)_ = 5.426, *p* < 0.001] and melody [*F*_(6, 128)_ = 12.55, *p* < 0.000001], but not of their interaction [*F*_(24, 504)_ = 1.060, *p* = 0.39]. Figure 2 gives an overall impression about the range of groove ratings across melodies and the size of the trends for the transforms, where melodies 1–5 are the simple ones and 6–12 the complex traditional songs. It can be seen that the original quantized versions always have the lowest ratings, and that 16D8A and 16D16A transformations are most often at the top. The *post-hoc p*-values in the above analysis were *p* < 0.05.

**Figure 3:**
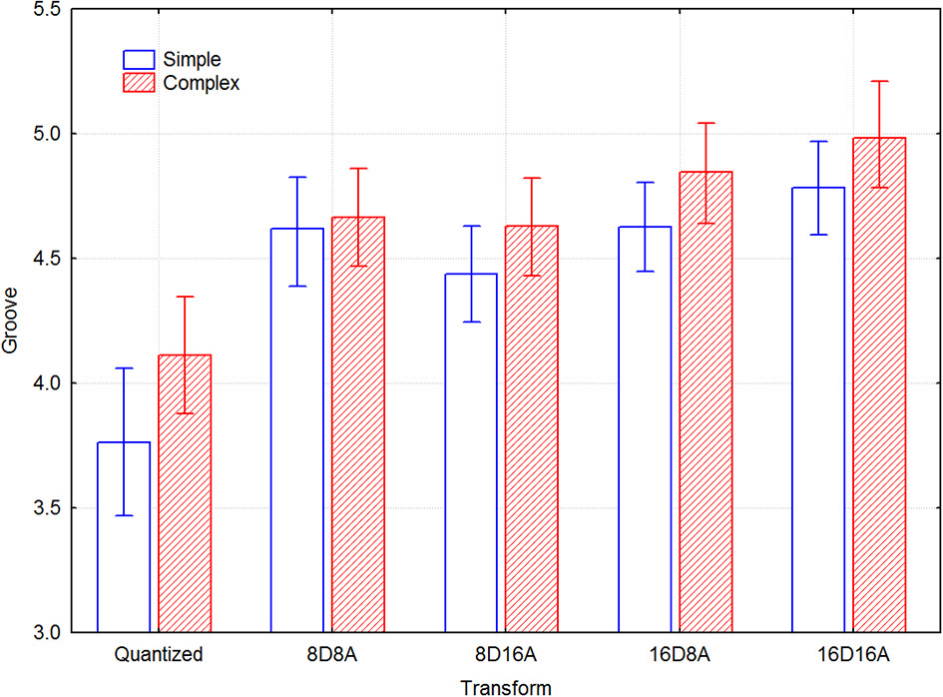
**Mean groove ratings for transform and melody type across participants**.

There are some differences in magnitude between the transforms for the two types of melodies, as indicated by the confidence intervals in Figure 3. Significant differences in the groove ratings can be seen between the transformations where the mean value of groove of one transformation does not overlap with the confidence interval of another transformation. The simple melodies generally have lower ratings, but they are increased more by the transformations than the complex melodies. For the transformed versions, there are only small differences between the simple and complex melodies. The only difference that seems to be significant is that for the complex melodies the 16DxA note transformations are somewhat more effective than the 8DxA transformations.

Table 1 lists the effect sizes (Cohen, 1988) for each transformation and melody type, which shows that Cohen's *d'* varies between 0.2 and 0.35.

**Table 1:** **Effects sizes for each transform relative to Quantized, separate for simple and complex melodies**.

**Transform**	**Melody type**	
	**Simple *N* = 110**	**Complex *N* = 154**
8D8A	0.295	0.221
8D16A	0.228	0.204
16D8A	0.306	0.283
16D8A	0.347	0.337

*All differences are significant (t = 2.71–4.19, df = 218/301, p < 0.01)*.

## Experiment 2

### Methods

**Figure 4:**
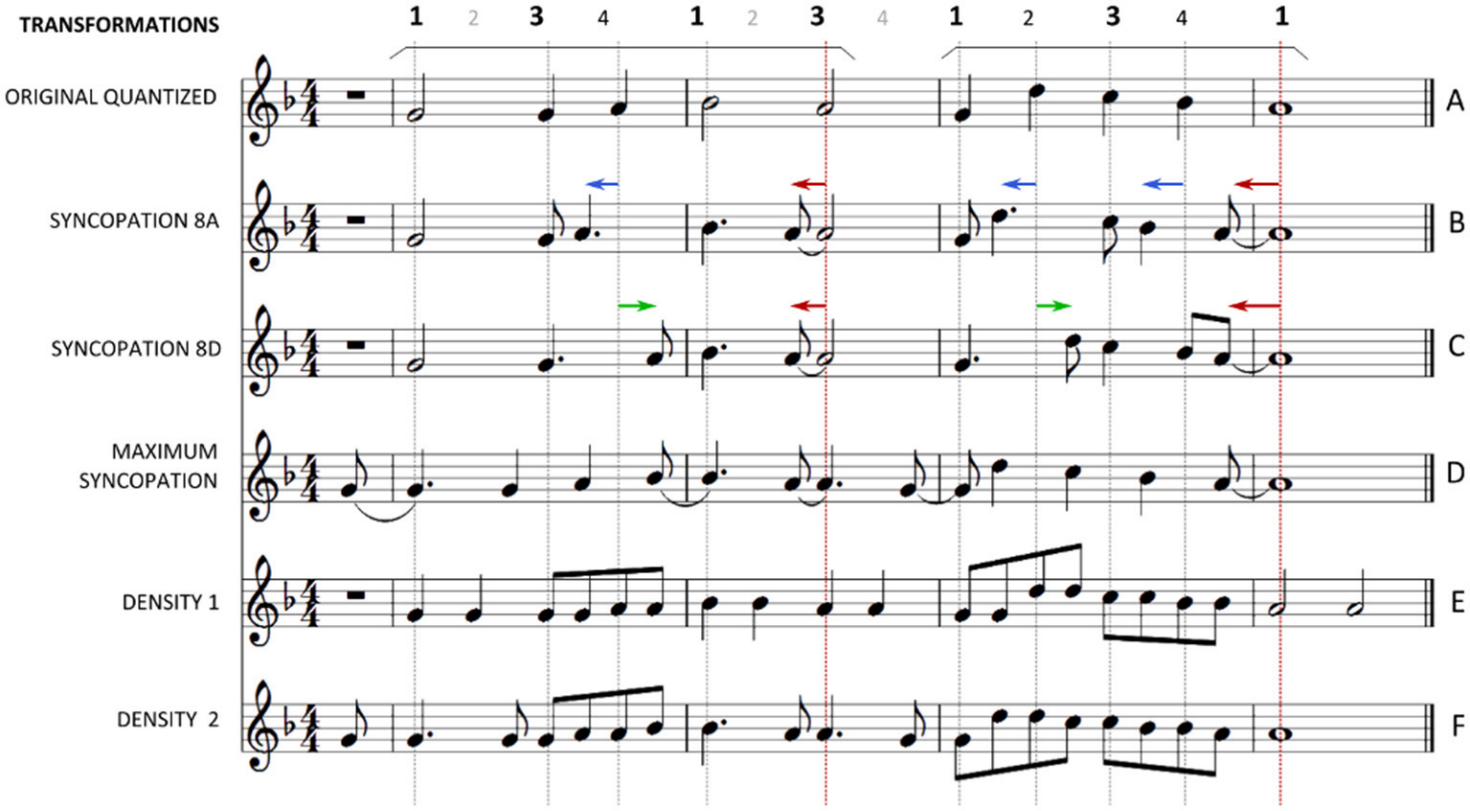
**Example of a piano melody and all the applied transformations of experiment 2**. The beats are annotated above the staffs. **(A)** The original quantized melody. An arch marks the duration of each phrase of the melody (in this example: the first two bars and the last two bars). **(B)** *Transformation 8A*. The last “strong” note of each phrase is anticipated by an 8 note. The “weak” quarter notes are anticipated by an 8 note. **(C)** *Transformation 8D*. The last “strong” note of each phrase is anticipated by an 8 note. The “weak” quarter notes are delayed by an 8 note. **(D)** *Maximum syncopation*. All notes have been anticipated by an 8 note. **(E)** *Density1*. All note durations have been divided in two thus duplicating each note. **(F)** *Density2*. Before each note of the original melody, a new note of the same pitch is introduced.

Experiment 1 showed that the syncopation transformations increased the groove ratings of the simple melodies, but also that all transformations had a similar effect. This raises the question whether any kind of transformation that introduces faster metrical levels but preserves the structure of the melodies would in fact result in higher ratings from the metronomic deadpan original version, or if syncopation is indeed required. Such an example would be a transformation of the original quantized melody that preserves the order and pitch of the note events as well as the original meter and phrase boundaries (see Figure 4, staffs **E,F**).

In Experiment 2 we aim to address this main question by including other non-syncopation transformations.

Additionally, we employ a set of three syncopation transformations that vary in the degree and strength of the syncopation that they generate. We wanted, in that way, to examine in more detail how syncopation affects the sensation of groove. In order to be able to achieve a higher degree of syncopation without altering the perceived meter, a metronome was employed to provide a strong metrical reference.

#### Participants

Twenty-two participants (6 female, 16 male) took part in the experiment (mean = 30.7 years, *SD* = 6.3 years). Of the 22 participants, six of them had no music training and nine of them were considered professional musicians with more than 8 years of music training. The participants were recruited via email, did not participate in experiment 1 and did not receive any remuneration for their participation

#### Stimuli

Five of the traditional complex songs from Experiment 1 and all five simple melodies that were composed for the purposes of Experiment 1 were used. A set of transformations was then applied resulting in 6 versions of each. The meter was emphasized by a hi-hat drum sound on every quarter note with the first quarter note of each bar being dynamically stressed (twice the MIDI velocity value). An introductory bar with only the metronome sound preceded the playback of each melody.

We wanted to compare the effect of the syncopation on the sensation of groove against the effect of transformations that did not introduce syncopation and preserved the main rhythmical and structural characteristics of the original melodies like the meter and phrase boundaries. To this end, we applied two kinds of transformations: (1) shifting note onset positions to introduce syncopation (Figure 4, staffs **B,C,D**) and (2) density transformations that doubled the number of notes per bar in the melodies without introducing syncopation (Figure 4, staffs **E,F**). In total, 6 different versions of each melody were generated: the original, three syncopated versions and two with increased density. Before applying any syncopation transformations, the melodies were manually segmented into short phrases of 2–4 bars as in Experiment 1. The transformations were then applied on each melodic phrase separately.

While the original version of each melody contained only slower metrical levels, i.e., only quarter notes (q.n. = 500 ms), half notes (1000 ms) and whole notes (2000 ms), applying any of the transformations introduced the faster 8th note metrical level (250 ms) but no 16th notes. A detailed description of the transformations follows.

The MIDI files of all melodies and their respective variations are available as Supplementary material.

***Syncopation transformation 8A***. The 8A transformation introduced a syncopating event at the end of each phrase (Figure 4B) by anticipating the last note of each phrase by an 8 note. Similar to the transformations of Experiment 1 the perceived meter was not affected since the remainder of the notes on strong beats, i.e., all notes on the first and third quarter note of each bar besides the last one of each phrase, were kept in their original positions. In addition to this end phrase anticipation, we generated additional syncopation by anticipating the notes found on the weak beats in each phrase, i.e., on the second and fourth quarter note of each bar, thus not affecting the perceived meter. Those syncopations are felt less strongly since they involve faster and weaker metrical levels (Longuet-Higgins and Lee, 1984), i.e., the quarter note metrical subdivision instead of the half bar or the bar.

***Syncopation transformation 8D***. The 8D transformation is identical to the 8D8A transformation of Experiment 1.

***Maximum syncopation***. The aim of the third syncopation transformation was to create a greater amount of syncopation. To this end, all notes in each original melody were anticipated by an 8 note. Although such a transformation shifts the entire metrical perception, syncopation was produced through the phase offset between the melody and the metronome.

***Density transformations***. Two density transformations were applied. In *density 1*, new notes were introduced after the original ones (Figure 1, staff **E**), and in *density 2*, new notes were introduced before the original ones (Figure 1, staff **F**).

***Density 1***. The density transformations doubled the density of note events per bar by introducing new notes of the same pitch as the original notes. The *density 1* transformation introduced after each note event in the original melody a new one of the same pitch, effectively halving each duration (Figure 1, staff **E**). For example, each quarter note became two 8 notes, each half note became two quarter notes etc. Since the original melodies contained no syncopation, the density transformation did not introduce syncopation, because all newly introduced notes on weak metrical positions were followed by the original notes of the melodies articulated in the strong metrical positions.

***Density 2***. The *density 2* transformation doubled the number of note events by introducing an 8 note before each note in the original melody (Figure 1, staff **F**). The introduced note had the same pitch as the original note following it. One can think of the transformation as the superposition of the original melody and the *maximum syncopation* transformation. Just as with the *density 1* transformation, no syncopation was introduced.

#### Rating scales

The rating scales were identical to those used in Experiment 1.

#### Design

The dependent variable was the rating of groove and the independent variables were (1) the type of transforms of the melodies (6 levels including 1 quantized, 2 density, and 3 syncopation), (2) melody type (simple or complex), and (3) the melodies themselves. A mixed design was employed such that two groups of participants rated half the conditions each, in order to make the task less taxing. Group 1 were given simple melodies #1 and #2 and the complex melodies #6, #7, and #8, and group 2 were given simple melodies #3, #4, and #5 and the complex melodies #9 and #10. The 30 within-participants conditions (5 melodies × 6 transforms) were presented in a different random order to each participant. Participants were required to listen to the whole recording before entering their ratings.

#### Implementation of the music examples

The implementation of the music examples was identical to those in Experiment 1, except for the addition of the metronome backing.

#### Procedure

The experiment was conducted online using a web-based interface, first developed for use in Davies et al. (2013). The user interface provided instructions for the participants to take the experiment in one session in a quiet listening environment, using either high quality headphones or loudspeakers. After reading the instructions and in order to proceed to the experiment, the participants consented to take part in the experiment. In addition, they entered some personal information including their sex, age and the number of years of musical training.

To allow participants to set the playback volume to a comfortable level, become familiar with the type of stimuli and to acquaint themselves with the functionality of the interface, a short training phase was conducted prior to the start of the main experiment. After the training phase, participants proceeded to the main experiment, during which their ratings were recorded. To reduce the time needed for participants to complete the experiment, the stimuli were split into two groups of equal size as described in Section Design. Upon completion of the main experiment, participants were able to offer feedback and comments which were recorded along with their ratings.

### Results

**Figure 5:**
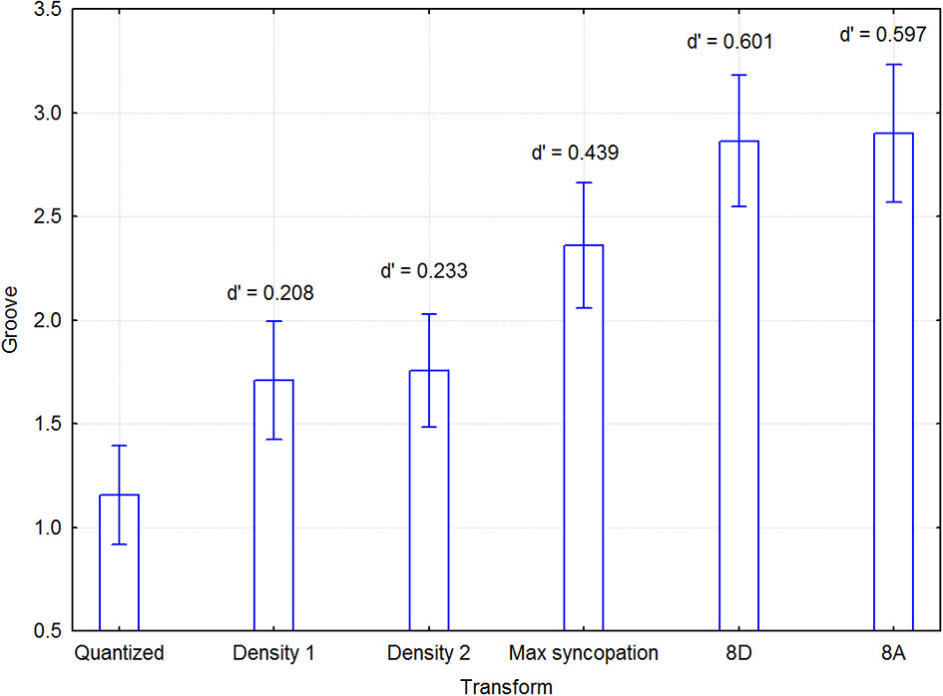
**Mean groove ratings for each transform, across participants and melodies (including melody type)**. Effect sizes for the differences between quantized and the syncopated transforms were quite respectable, and amounted to 0.208 for Density 1, 0.233 for Density 2, 0.439 for MaxSync, 0.601 for 8D, and 0.597 for 8A.

As described before, the melodies were distributed among two groups of participants so as to reduce the workload of each individual. This division was made to diffuse possible group effects, rather than to examine group x melody interactions. Since there were 5 simple and 5 complex melodies, each of these sets was therefore broken up in 2 and 3 melodies that were assigned to each group as described in Section Design. A mixed model 5 melody × 6 transformations within × 2 listener group between 3-Way ANOVA was initially conducted to examine possible effects of group. Melody type (simple vs. complex) was excluded from the ANOVA model because it rendered incomplete cells and unequal cell sizes. There were main effects of group [*F*_(1, 38)_ = 4.20, *p* = 0.047] and transform [*F*_(5, 190)_ = 22.74, *p* < 0.000001], but no interaction effects, notably not for any one involving group, such as group × melody [*F*_(3, 152)_ = 1.25, *p* = 0.293], group × transform [*F*_(5, 190)_ = 1.37, *p* = 0.237], or group × melody × transform [*F*_(20, 160)_ = 1.03, *p* = 0.422]. Thus, the only significant effect of group was that ratings were generally higher in group 1 (2.505 vs. 1.743, *N* = 1200, pooled *SD* = 2.186). Since this effect cannot alter the effect of the transform in any appreciable way, the data from both groups were aggregated. This means that we cannot determine to what extent the group difference emanated from the participants or the melodies, but this is of no consequence as melodies were not part of any hypothesis. A within-participants 2 Melody type × 5 Melody × 6 Transformations 3-Way ANOVA was conducted with groove as the dependent variable. The unit of analysis were pairs of participants, since melodies were originally distributed across two groups, and were entered as a random-effects variable. Main effects were found for Transformations [*F*_(5, 95)_ = 5.459, *p* < 0.0005] and Melody [*F*_(4, 76)_ = 97.37, *p* < 0.000001], but not for Melody type [*F*_(1, 19)_ = 0.686, *p* = 0.42]. As expected from the small Melody type effect, no effects involving this variable were significant except the 3-Way Transformations × Melody type × Melody interaction [*F*_(20, 380)_ = 1.964, *p* < 0.01]. In addition, the Transformations x Melody interaction was also significant [*F*_(20, 380)_ = 2.418, *p* < 0.001]. Melody did not relate to any linear dimension, but was included to pick up whatever systematic variance that happened to concur with the numbering of the melodies. Figure 5 shows that the two sparse syncopation transformations were the most effective. Significant differences in the groove ratings are indicated by the confidence intervals in Figure 5. For example, the *max syncopation* transformation has a significant difference in the groove ratings from all other transformations, while the mean rating of the *density 1* transformation is not significantly different from that of *density 2*. The *post-hoc p*-values in the above analysis were *p* < 0.05

Effect sizes for the differences between quantized and the transformations were quite respectable, and amounted to 0.208 for Density 1, 0.233 for Density 2, 0.439 for MaxSync, 0.601 for 8D, and 0.597 for 8A.

## Discussion

The purpose of the current study was to examine the effect of syncopation on the perception of groove independently of other expressive features of music performances. To this end, piano melodies were used that were transformed by an algorithm designed to introduce syncopation in a systematic and automatic way. We hypothesized that the syncopation transformations would result in higher groove ratings than the deadpan versions of the melodies with no syncopation. Additionally, we predicted that a higher number of introduced instances of syncopation would not have a proportional effect in the perception of groove and that the relation between syncopation and groove was complex—in the sense that it is not simply the amount of syncopation, but how it is expressed that causes the increase in the sensation of groove. In the simplest form this could follow an inverted U shape although an understanding of the relation between syncopation and groove requires a closer examination of the characteristics of individual instances of syncopation. All our hypotheses were verified in the experiments.

In Experiment 1, all transformations generated syncopation by anticipating the last note of each phrase in the melodies at the same time that they introduced faster metrical levels. The transformations displaced notes from the slower metrical levels, quarter note or half bar levels, to the faster 8th or 16th note levels generating a variety of combinations of note durations. However, this variety was not reflected in the groove ratings which were increased at similar levels for all the transformations.

In Experiment 2, we verified that the increase in the groove ratings was in fact particular to the syncopation, and that other types of transformations tended to have weaker effects on groove.

In addition, Experiment 2 examined whether the strength of the syncopation had an effect on groove. Our hypothesis was that a moderate number of syncopating notes that contradicted the meter only momentarily would increase the felt groove, while, in contrast, a high number of syncopating notes that constantly challenged the meter would not contribute to the groove perception. The hypothesis was tested by using two different strategies in generating syncopation. The first syncopation transformations, *8A* and *8D* transformations, syncopated certain notes while keeping most of the notes in place on the strong beats. In contrast, the *maximum syncopation* transformation displaced the entire melody and created a constant phase offset between the melody and the metronome sound. Both strategies resulted in higher groove ratings compared to the original versions, but less so for *maximum syncopation*.

Conversely, the *8A* and *8D* transformations did not differ in their respective groove ratings although they differ in the number of syncopating notes they generated. While *8D* generated only a single syncopating note at the strong beat at the end of each phrase, *8A* syncopated a substantial number of additional notes found on weak beats and that were simply delayed in *8D*. One possible explanation for this result is that the effect is due to the introduction of faster metrical levels and not specific to the syncopation. However, the two density transformations—which introduce the same faster metrical levels as the syncopation transformations without generating any syncopation—were rated significantly lower than the syncopation transformations.

Another explanation of the above results considers the nature of the syncopation in each transformation. In *8A* and *8D*, the meter is first clearly established and a strong beat sensation is created at the half bar metrical level. Then, at the end of each phrase, a single syncopating event violates our strong expectation that the last note should occur on the pre-established beat. The syncopation is felt very strongly because of the well-established beat and the position of syncopation in relation to the structure of the melody.

The “extra” syncopation in the *8A* version that results from the anticipation of the notes articulated on the weak quarter note level, i.e., on the second and fourth quarter note positions, is felt less strongly. The weak quarter note level comprises a subdivision of the more salient half bar pulse, which means that our expectation about events on these positions is accordingly lower. This is in accordance with several metrics of syncopation (Gómez et al., 2005) as well as with the recent study of Song et al. (2013) that points out the importance for the felt syncopation of the location of the individual instances of syncopation in relation to the meter. At the same time their position in each phrase results in grouping them together with the previous strong beat creating a Short–Long rhythmic figure with the short duration on the beat. The same kind of rhythmic figure is found in the *8D* variation but with the long note on the beat, resulting in no syncopation. This rhythmic figure repeats at least three times in most phrases before the final strong syncopation arrives. Together with the metronome and the harmonic structure of the melodies, these repetitions may enforce the meter, rather than contradict it, in both versions. Groove may therefore be affected only by the syncopating notes that clearly contradict our metrical expectation at metrically strong positions at the beat level and not by the weaker syncopation at the subdivision of the beat.

On the other hand, in the *maximum syncopation*, syncopation comes about through the interaction between the metronome and the melody. The melody, if it was to be heard alone, contains no syncopation. In this case, the syncopation cannot be attributed to individual syncopating events but to the interaction of rhythmic layers during the entire duration of the music, resulting in an overall feeling of syncopation with no distinct moments.

However, if the listeners ignored the metronome sound they could have perceived the melodies as non-syncopating and identical to the original versions. Although this would explain the lower ratings compared to the other syncopating versions, it would raise new questions about the higher ratings compared to the original versions or to those of the two density transformations. At the same time, the metronome sound with an emphasized downbeat was heard for one bar alone before each melody. On this basis, it seems unlikely that the listeners would ignore it the moment the syncopating melodies began. Then, even if, in the course of hearing the melody, they shifted their perception to align the meter to it, the contradiction with the stable metronome would persist, thus evoking a constant feeling of syncopation together with a strong sense of a stable meter and beat.

Hence, the ratings show that a moderate amount of syncopation arising from notable salient instances of syncopation that underline the melodic boundaries contribute more to the perception of groove than a higher degree of syncopation that is uniformly distributed along the entire duration and does not relate to the melodic structure. Most noticeably, the weakening of the groove sensation when the amount of syncopation is increased is not due to a breakdown of the perceived meter.

The results are compatible with the recent study by Witek et al. (2014) in which a moderate degree of syncopation was preferred to a high degree of syncopation. However, our current study differs significantly from Witek et al.'s. The synthesized stimuli were quite different in the two studies. Witek et al. used a number of real drum-breaks containing several timbres and each different example had a different degree of syncopation. In the present study, monophonic piano melodies that originally contained no syncopation were used, and syncopation was systematically generated by a computer algorithm resulting into different syncopated versions of the same melody. The simple rhythmic structure of the original melodies allowed for applying deterministic algorithms to manipulate their rhythmic characteristics. Introducing syncopation in already complex music could have a strong effect on the perceived meter, such as a shift of the downbeat. The automatic transformations of the experiments affected the particular rhythmic properties they were designed to change exclusively, that is, syncopation and density. More importantly, this method allowed us to draw specific conclusions about how syncopation can be applied effectively in order to create groove.

The syncopation transformations have the secondary effect of increasing the number of different note durations in the melodies. This is a direct consequence of their design. Excluding the final notes in each melody, the original melodies contained only two durations, which can be thought of as long (half notes) and short (quarter notes). The syncopation transformations in both experiments, with the exception of the *maximum syncopation* transformation, enrich the rhythms by introducing a variety of shorter and in-between short and long durations. The density and the *maximum syncopation* transformations do not have this effect. Although they all shorten the durations of the notes in the original melodies, they produce simple short–long relations. The result of Experiment 2 that the *maximum syncopation* is rated lower than the other two syncopated versions suggests that a variety in note durations could have a contribution to the sensation of groove. The transformations of experiment 1 introduce the various note durations gradually with the 8D16A and 16D8A resulting in higher variety than the 8D8A and 16D16A, due to the fact that they mix the 16 and 8 note metrical subdivisions. However, this is not reflected in the groove ratings that would be expected to be higher for the 8D16A and 16D8A if a direct relation between the variety in note durations and groove perception existed. Furthermore, despite the density 1 transformation generating greater number of durations and faster metrical levels compared to maximal syncopation—although much less compared to the 8D or 8A—, maximal syncopation was rated higher than density 1.

The relation between the diversity in note durations or the articulation of faster metrical levels in rhythms and groove perception therefore needs to be further explored as the results of the current study are inconclusive on this question. Nevertheless, the present study provides a systematic method for designing algorithms that can produce versions of melodies that contain faster and slower metrical levels as well as various degrees and diversity of note durations and different distributions of notes among those durations. It provides a method for directly comparing the different versions and examining them against listener ratings.

In conclusion, syncopation alone was sufficient to increase groove in simple melodies. We confirmed that a moderate amount of syncopation is more effective in creating the sensation of groove than a very high amount. However, the amount of syncopation, as a scalar quantity, that characterized each example was not sufficient to explain how syncopation increased the sensation of groove. To further explore this relation, an analysis of the individual instances of syncopation in the music examples was needed. This analysis showed that individual instances of syncopation that momentarily created rhythmic tension were more important in groove than an overall higher degree of syncopation. Our maximum syncopation condition, even though it increased the syncopation did not cause the breakdown of the perceived meter despite constantly challenging it. Furthermore, a richer rhythmic structure in note durations and metrical levels was considered a possible explanation for the relation between syncopation and groove as syncopation is inherently related to such structures. However, no definite conclusion was possible based on the results of the experiments.

### Conflict of interest statement

The authors declare that the research was conducted in the absence of any commercial or financial relationships that could be construed as a potential conflict of interest.

## Footnotes

^1^http://www.native-instruments.com/en/products/komplete/samplers/kontakt-5/overview/library/

## References

[B1] Cohen J. (1988). Statistical Power Analysis for the Behavioral Sciences. Hillsdade, NJ: Routledge

[B2] Davies M. Madison G. Silva P. Gouyon F. (2013). The effect of microtiming deviations on the perception of groove in short rhythms. Music Percept. 30, 497–510 10.1525/mp.2013.30.5.497

[B3] Gómez F. Melvin A. Rappaport D. Toussaint G. T. (2005). Mathematical measures of syncopation, in Proceedings of the BRIDGES: Mathematical Connections in Art, Music and Science (Banff, AB), 73–84

[B4] Gómez F. Thul E. Toussaint G. T. (2007). An experimental comparison of formal measures of rhythmic syncopation, in Proceedings of the International Computer Music Conference (Copenhagen), 101–104

[B5] Huron D. (2006). Sweet Anticipation: Music and the Psychology of Expectation. Cambridge, MA; London: The MIT Press

[B6] Huron D. Ommen A. (2006). An empirical study of syncopation in American popular music, 1890-1939. Music Theory Spectr. 28, 211–231 10.1525/mts.2006.28.2.211

[B6a] Janata P. Tomic S. T. Haberman J. M. (2012). Sensorimotor coupling in music and the psychology of the groove. J. Exp. Psychol. Gen. 141, 54–75 10.1037/a0024208 21767048

[B7] Keil C. (1995). The theory of participatory discrepancies: a progress report. Ethnomusicology 39, 1–19 10.2307/852198

[B8] London J. (2012). Hearing in Time. 2nd Edn New York, NY: Oxford University Press 10.1093/acprof:oso/9780199744374.001.0001

[B9] Longuet-Higgins H. C. Lee C. S. (1984). The rhythmic interpretation of monophonic music. Music Percept. 1, 424–441 10.2307/40285271

[B10] Madison G. (2006). Experiencing groove induced by music: consistency and phenomenology. Music Percept. 24, 201–208 10.1525/mp.2006.24.2.201

[B11] Madison G. Gouyon F. Ullén F. Hörnström K. (2011). Modeling the tendency for music to induce movement in humans: first correlations with low-level audio descriptors across music genres. J. Exp. Psychol. Hum. Percept. Perform. 37, 1578–1594 10.1037/a0024323 21728462

[B12] Madison G. Sioros G. (2014). What musicians do to induce the sensation of groove in simple and complex melodies, and how listeners perceive it. Front. Psychol. 5:894 10.3389/fpsyg.2014.00894 25191286 PMC4137755

[B13] Povel D.-J. Okkerman H. (1981). Accents in equitone sequences. Percept. Psychophys. 30, 565–572 10.3758/BF03202011 7335453

[B14] Sioros G. Guedes C. (2011). Complexity driven recombination of MIDI Loops, in Proceedings of the 12th International Society for Music Information Retrieval Conference (Miami, FL), 381–386

[B15] Sioros G. Miron M. Cocharro D. Guedes C. Gouyon F. (2013). Syncopalooza: manipulating the syncopation in rhythmic performances, in Proceedings of the 10th International Symposium on Computer Music Multidisciplinary Research (Marseille: Laboratoire de Mécanique et d'Acoustique), 454–469

[B16] Song C. Simpson A. J. R. Harte C. A. Pearce M. T. Sandler M. B. (2013). Syncopation and the score. PLoS ONE 8:e74692 10.1371/journal.pone.0074692 24040323 PMC3769263

[B17] Temperley D. (1999). Syncopation in rock: a perceptual perspective. Pop. Music 18, 19–40 10.1017/S0261143000008710

[B18] Witek M. A. G. Clarke E. F. Wallentin M. Kringelbach M. L. Vuust P. (2014). Syncopation, body-movement and pleasure in groove music. PLoS ONE 9:e94446 10.1371/journal.pone.0094446 24740381 PMC3989225

